# HSV-1 latent rabbits shed viral DNA into their saliva

**DOI:** 10.1186/1743-422X-9-221

**Published:** 2012-09-28

**Authors:** James M Hill, Nicole M Nolan, Harris E McFerrin, Christian Clement, Timothy P Foster, William P Halford, Konstantin G Kousoulas, Walter J Lukiw, Hilary W Thompson, Ethan M Stern, Partha S Bhattacharjee

**Affiliations:** 1 Department of Ophthalmology LSUHSC School of Medicine, 533 Bolivar Street, Room 3D13, New Orleans, LA, 70112, USA; 2 Department of Pharmacology, LSUHSC, 1901 Perdido Street, New Orleans, LA, 70112, USA; 3 Department of Microbiology, LSUHSC, 1901 Perdido Street, New Orleans, LA, 70112, USA; 4 Neuroscience Center, LSUHSC, 2020 Gravier Street, 8th Floor, New Orleans, LA, 70112, USA; 5 Tulane University, 6823 St Charles Avenue, New Orleans, LA, 70118, USA; 6 Department of Biology, Xavier University of Louisiana, One Drexel Drive, New Orleans, LA, 70125, USA; 7 Department of Microbiology and Immunology, SIU School of Medicine, 825 North Rutledge St., Room 2668, Springfield, IL, 62702, USA; 8 Department of Pathobiological Sciences, School of Veterinary Medicine, LSU, Skip Bertman Drive, Baton Rouge, LA, 70803, USA; 9 Department of Biostatistics, School of Public Health, 2020 Gravier Street, 3rd floor, New Orleans, LA, 70112, USA

**Keywords:** HSV-1, Rabbit, Saliva, Tears, Spontaneous HSV-1 shedding, Real-time-PCR

## Abstract

**Background:**

Rabbits latent with HSV-1 strain McKrae spontaneously shed infectious virus and viral DNA into their tears and develop recurrent herpetic-specific corneal lesions. The rabbit eye model has been used for many years to assess acute ocular infections and pathogenesis, antiviral efficacy, as well as latency, reactivation, and recurrent eye diseases. This study used real-time PCR to quantify HSV-1 DNA in the saliva and tears of rabbits latent with HSV-1 McKrae.

**Methods:**

New Zealand white rabbits used were latent with HSV-1 strain McKrae and had no ocular or oral pathology. Scarified corneas were topically inoculated with HSV-1. Eye swabs and saliva were taken from post inoculation (PI) days 28 through 49 (22 consecutive days). Saliva samples were taken four times each day from each rabbit and the DNA extracted was pooled for each rabbit for each day; one swab was taken daily from each eye and DNA extracted. Real-time PCR was done on the purified DNA samples for quantification of HSV-1 DNA copy numbers. Data are presented as copy numbers for each individual sample, plus all the copy numbers designated as positive, for comparison between left eye (OS), right eye (OD), and saliva.

**Results:**

The saliva and tears were taken from 9 rabbits and from 18 eyes and all tested positive at least once. Saliva was positive for HSV-1 DNA at 43.4% (86/198) and tears were positive at 28.0% (111/396). The saliva positives had 48 episodes and the tears had 75 episodes. The mean copy numbers ± the SEM for HSV-1 DNA in saliva were 3773 ± 2019 and 2294 ± 869 for tears (no statistical difference).

**Conclusion:**

Rabbits latent with strain McKrae shed HSV-1 DNA into their saliva and tears. HSV-1 DNA shedding into the saliva was similar to humans. This is the first evidence that documents HSV-1 DNA in the saliva of latent rabbits.

## Background

The rabbit eye model has been used for HSV-1 studies since 1960 [1-7]. One of the first studies in 1965 on HSV-1 latency in the rabbit eye model was by Laibson and Kibnick [4]. Since 1978 [8], we have utilized the rabbit eye model for HSV-1 studies on antiherpetic chemotherapy [8-13], HSV-1 latency [14,15], and spontaneous and induced viral reactivations and recurrent ocular herpetic disease [16-35]. We have also investigated up-regulation and down-regulation of host gene expression [36,37] and alterations in reactivation phenotypes in HSV-1 genomic structure by histone modifications as a result of mutations in the viral genome [38-41] that take place following induction stimuli that could induce reactivation [42-44].

Our previous studies [45-51] have focused on the cornea, tears, and trigeminal ganglia [52-54]. In this report, we document for the first time the detection of HSV-1 DNA in the saliva of rabbits latent with HSV-1 McKrae. The McKrae strain is known to be a high phenotypic reactivator in the rabbit eye model [24,53-55]. HSV-1 DNA and infectious virus from saliva of patients have been reported in many studies, the first of which appeared in 1953. We have reviewed the human studies on saliva, 10 studies on infectious virus, and 18 studies in HSV-1 DNA (Tables 1[56-64] and 2[65-77]). Miller and Danaher reviewed most of those cited **[**[78]. The “gold” standard of proof of HSV-1 is detection of infectious virus. The sensitivity of the PCR, as well as the increase in frequency of sampling per day, has increased the percent of positives of HSV-1 and HSV-2 to almost 100% of humans tested [73,77,79-81]. 

**Table 1:** **Asymptomatic shedding of infectious HSV-1 detected by cell culture from mouth swabs of healthy subjects**

**Author(s), Year**	**N**	**Individuals: total positive/total subjects**	**Shedding frequency: total positiveswabs/total swabs**	**Mouth swabs frequency: numberof swabs/subject**
Buddingh et al., 1953 [56]	368*	30/368 (8.2%)	30/368 (8.2%)	one
Buddingh et al., 1953 [56]	185**	5/185 (2.4%)	5/185 (2.7%)	one
Kaufman et al., 1967 [57]	35	6/35 (17.1%)	6/700 (0.9%)	20
Lindgren et al., 1968 [58]	418	8/418 (1.9%)	8/2204 (0.4%)	5-6
Douglas & Couch, 1970 [59]	10	8/10 (80%)	11/494 (2.2%)	~49
Hatherley et al., 1980 [60]	384	37/384 (9.6%)	47/1536 (3.1%)	4
Spruance, 1984 [61]	8	8/8 (100%)	47/637 (7.4%)	105
Kameyama et al., 1988 [62]	110	5/110 (4.5%)	70/7805 (0.9%)	71
Tateishi et al., 1994 [63]	1000	27/1000 (2.7%)	27/1000 (2.7%)	one
Okinaga, 2000 [64]	10	4/10 (40%)	4/870 (0.5%)	87
**Total**	2528	138/2528 (5.5%)	255/15799 (1.6%)	

*adults.

**children.

**Table 2:** **Asymptomatic shedding of HSV-1 detected by PCR from mouth swabs (saliva) of healthy subjects**

**Authors, Year**	**N**	**Individuals: total positive/total subjects**	**Shedding frequency: total positiveswabs/total swabs**	**Mouth swab frequency: numberof swabs/subject**
Robinson et al., 1992 [65]	12	12/12 (100%)	0/0 (0%)	0
Robinson et al., 1992 [65]	12*	0/12 (0%)	0/0 (0%)	0
Tateishi et al., 1994 [63]	1000	46/1000 (4.6%)	46/1000 (4.6%)	one
Kriesel et al., 1994 [66]	27	3/27 (11%)	3/27 (11%)	one
Lee et al., 1996 [67]	87	12/87 (13.8%)	12/87 (13.8%)	one
Knaup et al., 2000 [68]	30	19/30 (63.3%)	39/290 (13.5%)	9-10
Druce et al., 2002 [69]	477	43/477 (9%)	43/477 (9%)	one
Gleeson et al., 2002 [70]	14	0/14 (0%)	0/0 (0%)	0
Youssef et al., 2002 [71]	5	1/5 (20%)	1/5 (20%)	one
Miller et al., 2004 [72]	123	11/123 (8.9%)	11/123 (8.9%)	one
Miller et al., 2004 [72]	63**	13/63 (21%)	17/217 (7.8%)	3-4
Kaufman et al., 2005 [73]	50	46/50 (92%)	1020/2712 (37.6%)	54
Miller et al., 2005 [74]	58	1/58 (1.7%)	1/58 (1.7%)	one
da Silva et al., 2005 [75]	25	25/25 (100%)	162/309(52.4%)	12-13
Lin et al., 2005 [76]	60	4/60 (6.7%)	4/60 (6.7%)	one
Kumar et al., 2009 [77]	14*****	12/14 (86%)	357/840 (42%)	60
Kumar et al., 2009 [77]	15***	15/15 (100%)	465/900 (52%)	60
Kumar et al., 2009 [77]	16****	15/16 (94%)	701/961 (73%)	60
**Totals**	2076	278/2088 (13%)	2882/8066 (36%)	

*Had no clinical history of HSV-1.

**Had dental procedure prior to mouth swab.

*** Received Valtrex treatment + Placebo treatment.

****Received Valtrex treatment + Aspirin treatment.

***** Received 2 Placebo treatments.

The Kumar et al., 2009 study was double blind. Placebo and control had trials in healthy adults.

## Results and discussion

Starting on PI day 28 and continuing for 22 consecutive days until PI day 49, saliva and tear swabs were taken. Real-time PCR was done on all of the saliva and tear samples. All nine rabbits shed HSV-1 DNA at least once in their tears and saliva (summarized in Table 3). Cumulative data showed 43.4% (86/198) of saliva samples were positive for HSV-1 DNA and 28.0% (111/396) of tear samples were positive.

**Table 3:** **Summary of data from saliva and tears**

**Characteristics**	**HSV-1 Strain McKrae**	
	**Saliva/ mouth**	**Tears/eyes (OD & OS)**
**Rabbits (+/total)**	9/9 (100%)	9/9 (100%)
**Swabs (+/total)**	9/9 (100%)	18/18 (100%)
**Total positive swabs/total swabs**	86/198	111/396
**Total positive swab (%)**	(43.43%)	(28.03%)
**Mean ± SEM copynumbers**	3773 ± 2019	2287 ± 868
**Lowest copy number**	28	20
**Highest copy number**	337,894	205,000
**Median copy number**	439	553
**Total number of episodes**	49	75
**Range of positives**	6-15	1-9
**Average positives/days** **(Total positives/days of swabs)**	86/22 = 3.9	111/44 = 2.5
**Average duration of shedding(Total positive/rabbit saliva orrabbit eyes)**	86/9 = 9.6 days	111/18 = 6.2 days

**Figure 1:**
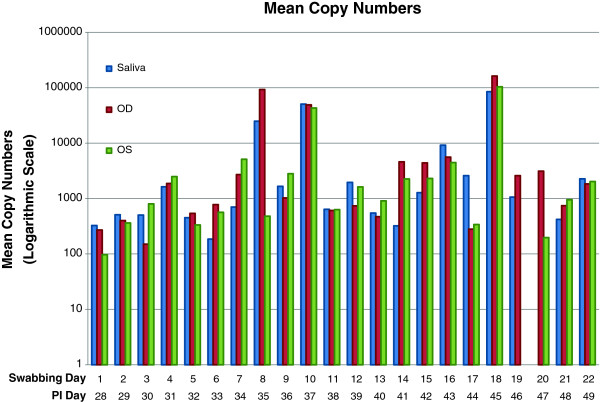
Mean copy numbers of HSV-1 DNA per day post inoculation, taken from left eye (OS), right eye (OD), and saliva of nine rabbits.

Figure 1 displays the copy numbers on a logarithmic scale obtained from left eye (OS), right eye (OD), and saliva for each day. The mean copy number ± the SEM of HSV-1 DNA in saliva was 3773 ± 2019 and for tears 2284 ± 868. The median copy number for saliva was 439 and the median copy number for tears was 553. The real-time PCR copy numbers for tears ranged from a low of 20 to a high 205,000. The real-time PCR copy numbers for saliva ranged from a low of 28 to a high 337, 900.

**Figure 2:**
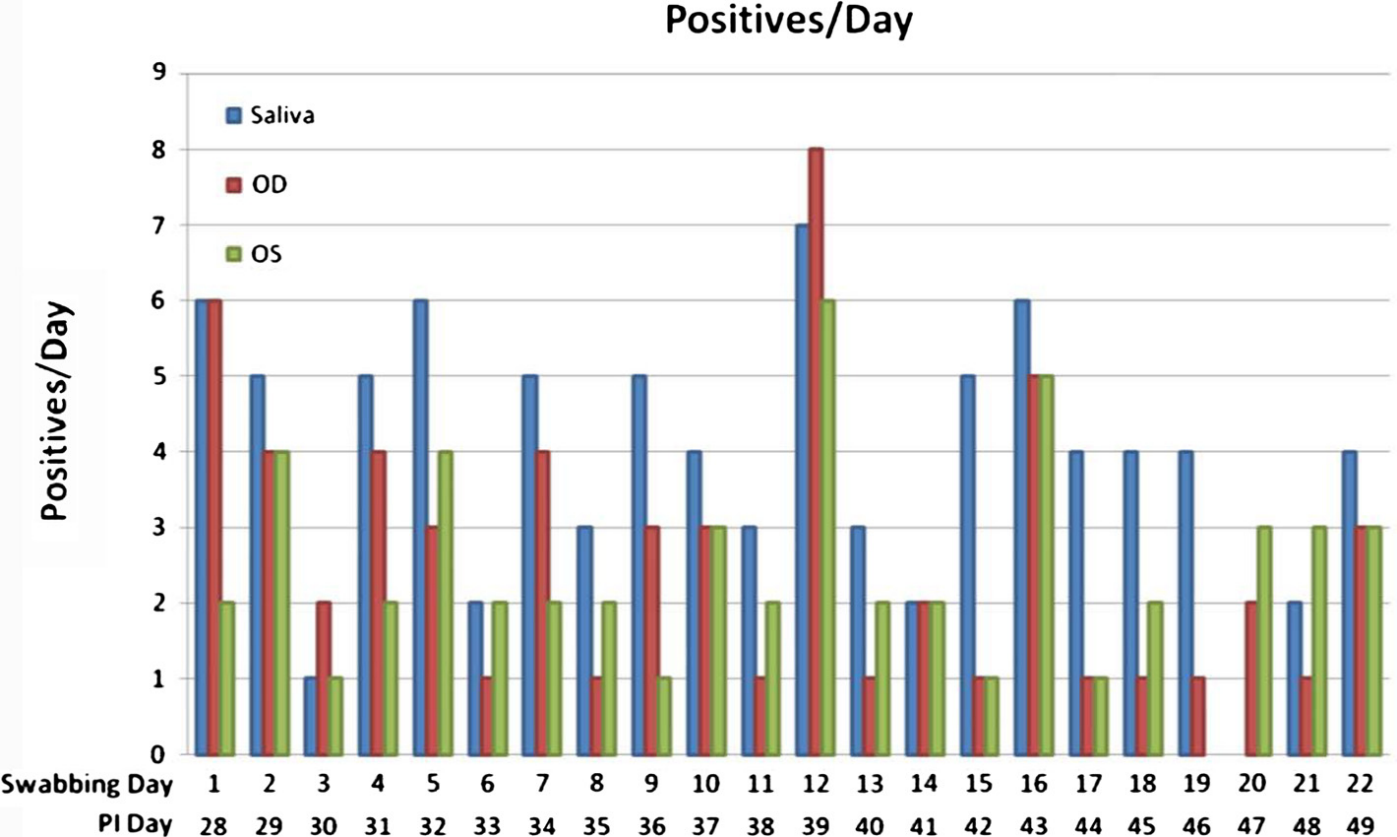
Number of positives per day post inoculation, taken from left eye (OS), right eye (OD), and saliva of nine rabbits.

Figure 2 displays data showing positives over totals from OD, OS, and saliva, for PI days 28 to 49. Episodes of shedding are defined by finding zero virus, followed by positive virus, followed by zero virus. There were 49 episodes of shedding in saliva and 75 episodes in tear swabs. The average duration of shedding for saliva was 9.6 (86/9) days and for eyes the average duration of shedding was 6.2 (111/18) days.

**Figure 3:**
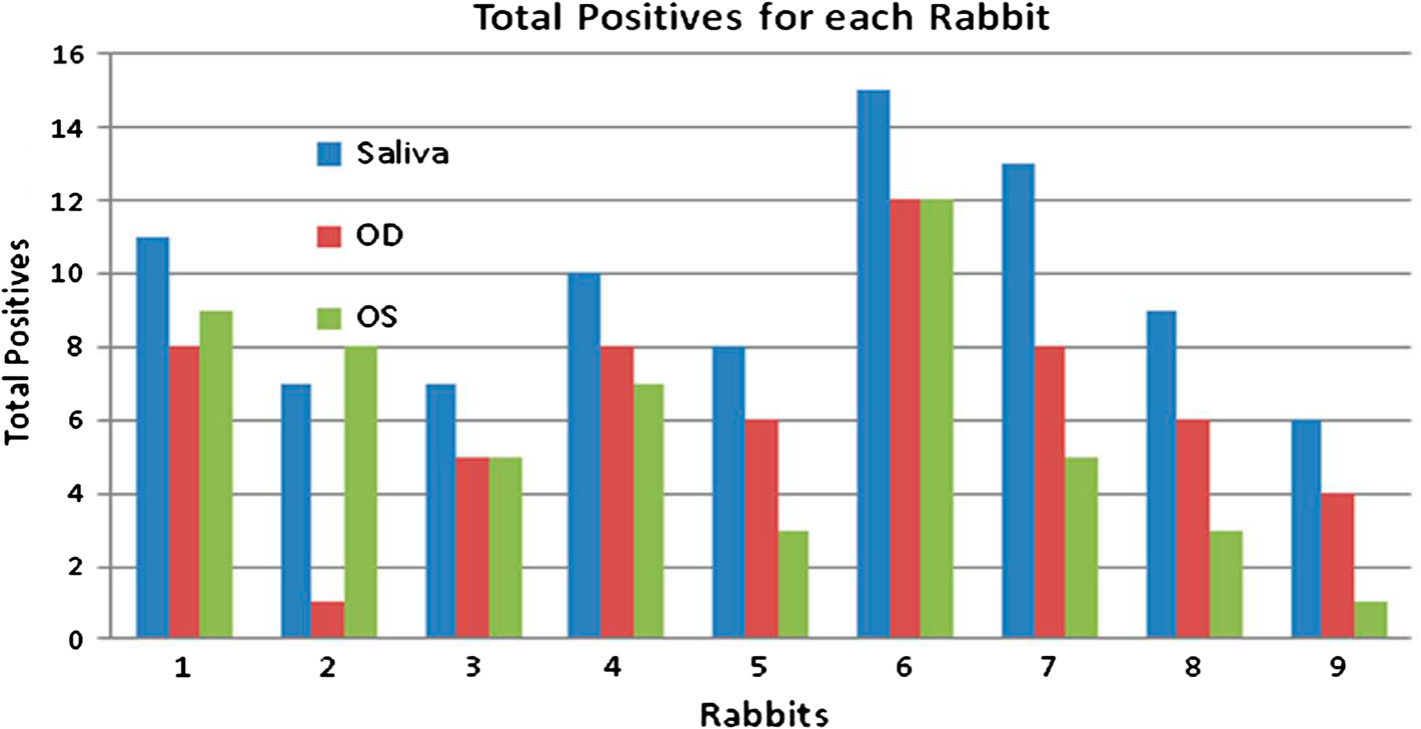
Total number of positives per rabbit, from left eye (OS), right eye (OD), and saliva.

Total positives from individual rabbits are shown in Figure 3. Each positive value is the total copy number by real-time PCR for each swab. Figure 3 provides data for saliva and for left and right eyes for each rabbit.

**Figure 4:**
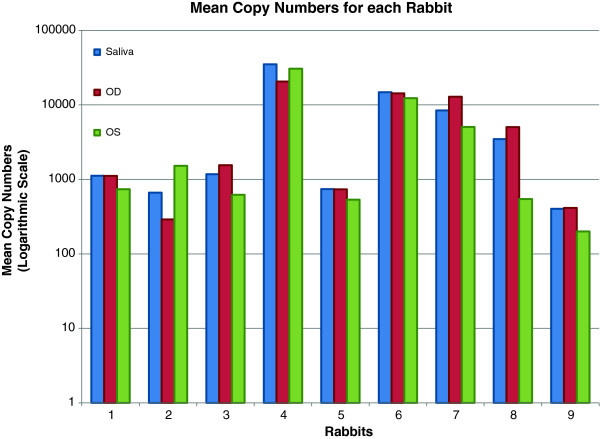
**Mean copy numbers of HSV-1 DNA for each rabbit for left eye (OS), right eye (OD), and saliva.** All rabbits tested positive for virus in both tears and saliva at least once.

Figure 4 shows the mean copy number for each of the 9 rabbits for saliva, right eye, and left eye.

We speculate that the potential source of HSV-1 that appears in the tears and saliva could be from many ganglia in the head and neck region. Richter et al [82]. assessed 8 different types of human ganglia in the head and neck regions of 36 formalin-fixed cadavers, assaying for HSV-1, HSV-2 and VZV from a total of 415 ganglia samples. For HSV-1, 36% (150/415) were positive; 89% (32/36) were positive in at least one sample. Certainly the TG could be the source of most of the virus that appears in the saliva and tears since the TG has been studied most frequently and is the largest ganglia in the head and neck region. However, we suggest, based on evidence from Shimeld et al [83] that the virus is not latent in a specific region for the ganglia but could be in all three regions, referred to as V1, V2, and V3.

The first isolation of infectious HSV-1 from the oral cavity (saliva) was in 1953 by Buddingh et al [56] took place at the LSU School of Medicine in New Orleans. Saliva was obtained from 571 healthy volunteers (386 children >15 years of age and 185 adults <15 years of age). The oral swabs were assayed using the chorioallantois of 12-day-old chick embryos. The results showed that infectious HSV-1 was detected at a rate three times greater in the saliva from children than adults. Although these experiments were conducted almost 60 years ago, comparison of these data with more recent studies from humans (see review [84]) demonstrates a strong general correlation [57] and attests to the efficiency of the HSV-1 detection system used by Buddingh et al [58].

The first report of detection of infectious HSV-1 in human tears by Kaufman et al. appeared in 1967 [57]. Our review article in 2009 [84] summarized the numerous reports of asymptomatic shedding of infectious HSV-1 from human tears and saliva. Also, our other reviews [53,54,85] have summarized results from our and other laboratories of the detection of infectious HSV-1 and viral DNA in tears from HSV-1 latent animals such as rabbits and mice.

The development of very sensitive real-time PCR for detection of HSV-1 DNA has resulted in many studies of tears from humans and animal eye models; however, we know of no reports of either infectious virus or HSV-1 DNA detected in the oral cavity (saliva) of animals latent with HSV-1.

This study used the McKrae strain of HSV-1 known to be a high phenotypic reactivator in rabbits that can cause spontaneous shedding into the tear film of infectious virus, HSV-1 DNA, and spontaneous development of HSV–specific corneal epithelial lesions. This is the first study that documents shedding of HSV-1 DNA in the saliva and compares that to the shedding of HSV-1 DNA that occurs in the tears of these latently infected rabbits.

HSV-1 strains that are high phenotypic reactivators, such as 17 Syn + and McKrae, are excellent for studying ocular latency, reactivation, and recurrent ocular diseases in the rabbit eye model. There are disadvantages of using these strains in rabbits: the mortality is approximately 45%, and 5-10% of the surviving rabbits have eyes with stromal opacity or that are otherwise not completely healed. We never use any eyes that are not completely healed. Thus, the ability to assess saliva has benefits and practicality when corneas are not completely healed.

The frequency (43.4%) of positives assessed by real-time PCR of the saliva was almost twice that of the positive tears (28.0%). Table 3 gives the average positives per days of swabs for the saliva as 3.9 (86/22). For both eyes, the average positive was 2.5 (111/44). The average duration of shedding for saliva is 9.6 days (86/9). For the 18 eyes, the average duration of shedding was 6.2 days (111/18). The HSV-1 copy numbers in the tears have a higher median copy number (533) compared to the saliva, which had a median copy number of 439. There was no statistical difference in the mean copy numbers from the saliva and tears. The frequency of coincidence of simultaneous shedding of the right eye, left eye, and saliva being positive was 12.6% (25/198). It is impractical to repeatedly swab the eyes, since this can induce epithelial trauma and neovascularization. As a result, saliva swabs were taken four times each day from each rabbit while only one swab was taken each day for tears. Higher frequency of positives from the saliva could be due to greater frequency of swabs or another explanation is that the saliva is a combination of the flow of tears into the oral cavity from both eyes as well as shedding of the virus from the nerve endings in the oral cavity. More studies with different strains of HSV-1 need to be conducted to determine patterns of HSV-1 copy numbers and frequencies (percent positives).

## Conclusions

The rabbit eye model has been used to investigate vaccines to prevent acute ocular herpes (prophylactically) or block recurrent ocular herpes (therapeutically) [86-90]. Many immunological and virological assays can be conducted to assess the efficiency of an HSV vaccine as to whether it is prophylactic and/or therapeutic. This new discovery that latent rabbits shed HSV-1 DNA in their saliva reliably and with very high frequency can be used to assess the efficacy of HSV-1 vaccines, as well as to determine the efficacy of antiviral chemotherapy. This discovery that rabbits latent with HSV-1 strain McKrae spontaneously shed viral DNA in their saliva significantly increases the utility and practicality of the rabbit model and highlights its similarity to humans.

## Methods

### Rabbits, cells, and virus

All experimental procedures were performed in accordance with the ARVO Resolution for the Use of Animals in Ophthalmic and Vision Research and were approved by the LSU Health Sciences Center (LSUHSC) Institutional Animal Care and Use Committee (IACUC #2850). New Zealand white (NZW) rabbits were obtained from McNeil Rabbitry (Moss Point, MS). The HSV-1 strain employed was McKrae (obtained as a low passage isolate from H.E. Kaufman). Strain McKrae was used to inoculate rabbit corneas. Before inoculation, numbers of plaque-forming units (PFU) were determined in a standard plaque assay procedure using CV-1 cells. All of the viral stocks were grown at a very low multiplicity of infection in primary rabbit kidney cells. Rabbits were equilibrated for seven days in the LSUHSC animal care facility prior to viral inoculation.

### Experimental design

For corneal inoculation, the rabbits were anesthetized by intramuscular administration of xylazine (6.6 mg/kg of body weight) and ketamine (100 mg/kg of body weight). The eyes were topically anesthetized with 1.0% tropicamide. The corneas were mildly scarified in a 2 × 2 cross-hatch pattern and each cornea was inoculated with 5 × 10^5^ PFU of strain McKrae. All corneas developed dendritic lesions as verified by slit-lamp examination. The rabbits were visually examined daily for health and ocular conditions for 27 days. The eyes were examined with a slit lamp on PI day 27 to verify the absence of ocular abnormalities; none were found in any rabbit used. Also, visual examinations by the LSUHSC veterinarian found no observable lip or oral lesions; thus, no rabbit had symptoms of HSV-1 infections in the head and neck region or any other areas on its body.

### Collection of saliva from HSV-1 latent rabbits

Saliva was collected on polyester swabs (Puritan Medical Products Co., LLC, Gilford, ME) from rabbits beginning on PI day 28 and continuing for 20 consecutive days. The saliva was collected each day in the early morning four consecutive times approximately 20–40 minutes between the repeated samples; thus each rabbit had four saliva swabs taken each day, which were combined so that there was one saliva sample per rabbit per day. All swabs were processed either on the day taken or within 72 hours. If not immediately processed, they were frozen at −80°C, until DNA was extracted similar to other studies that we have published [77].

### DNA elution of swabs of rabbit saliva

Each individual swab was processed as previously described [77] with the exception that prior to the supernate extraction and before precipitation, all samples were pooled for each day from each rabbit into one saliva sample. The DNA from the swabs was extracted with a DNA elution kit (Gentra Puregene; Qiagen Sciences, Germantown, MD), and the DNA samples were stored in DNA hydration buffer (provided with the kit) at 4°C until processed by real-time PCR. Sterile unused swabs were processed as a negative control. Other swabs were spiked with either infectious virus of each strain or purified HSV-1 DNA; these were processed as positive controls for DNA extraction by the same method noted above. We determined that the efficiency of extraction of HSV-1 particles and HSV-1 DNA and the efficacy ranged between 58-66%. Oral swabs from naïve rabbits were used as an additional negative saliva control.

### Collection of tears and DNA elution from eye swabs

The procedures and protocols used for these studies were the same as previously reported [15,73,77]. One swab was taken daily from each eye for 22 consecutive days.

### HSV-1 DNA quantification

HSV-1 copy numbers from the DNA samples were determined by calculating the number of DNA polymerase genes in the sample according to the previously described methods [15,77,91]. The sequences of forward and reverse primers were 5’-AGA GGG ACA113 TCC AGG ACT TTG T-3’ and 5’-CAG GCG CTT GTT GGGT GTA C-3’, respectively (Integrated DNA Technologies [IDT], Coralville, IA). The primer pair was synthesized by IDT. The probe was 5’6-FAM/ACC GCC GAA CTG AGC A/3’ BHQ-1 (IDT). All reactions were done in a total volume of 20 μl. The 20 μl of reaction mixture contained 1 TaqMan® Universal Mastermix (Applied Biosystems, Inc., Foster City, CA), 100 nM of primers and probe, and 5 μl of DNA sample. All reactions were performed in 96-well plates (Bio-Rad, Hercules, CA), which were centrifuged for 1 minute at 1000 *g* and room temperature in a swing-bucket rotor (CRU 5000 centrifuge; Damon/IEC, Needham, MA) to remove any air bubbles. The reaction conditions were as follows: 95°C for denaturation for 10 sec, 55°C for annealing for 30 sec, and 72°C extension for 10 sec in the real-time PCR (iCycler iQ; Bio-Rad) system for 45 cycle repeats. All samples were analyzed in triplicate. Only samples in which all three were determined to be positive were used. Each reaction plate contained both positive and negative controls as described above as well as HSV-1 DNA standards. The cosmid containing the HSV-1 DNA polymerase gene was obtained from Dr. David C. Bloom (University of Florida, Gainesville, FL) and used as a standard for this study. The cosmid contained a copy of the 4.8 kb restriction fragment (*Hin*dIIIA) encompassing the HSV-1 DNA polymerase gene from the HSV-1 strain 17Syn+. A standard curve was generated from both 10-fold and 2-fold serial dilutions of the p*Hin*dIIIA cosmid. The lowest dilutions assessed in the 10-fold and 2-fold series were below 1 copy.

### Statistical analysis

Standard statistical procedures were used to determine the mean, the median, and the SEM. Detailed statistics were used to determine *P*-values among the two groups (saliva vs. tears) for the HSV-1 DNA copy number and the total positives. Longitudinal series of observations were analyzed to include intersubject correlation. The data were analyzed by repeated-measures analysis of variance (ANOVA). Our main interest was the comparison of significance between the two groups to determine a between-subject effect. *P*-values given were those for the *F* test of the repeated measures ANOVAs. This analysis is similar to that done by Kumar et al [15,77].

## Abbreviations

HSV-1: Herpes simplex virus type 1; PI: Post-inoculation; RT-PCR: Real-time-polymerase chain reaction; HPR: High phenotypic reactivator.

## Competing interests

The authors have no competing interest in anything mentioned in this article.

## Authors’ contributions

All authors collaborated on the experimental design, the calculation and preparation of tables and figures, and discussion of the experimental results. JMH conceived the basic strategic plan. All authors participated in the manuscript preparation. NMN, CC, HEM, and PSB participated in the inoculations, slit lamp experiments, and collections of eye swabs and saliva and performing the real-time PCR. HWT did statistical analyses.
